# Effects of Alkali on Water Soluble Hexavalent Chromium in Ordinary Portland Cement

**DOI:** 10.3390/ijerph19084811

**Published:** 2022-04-15

**Authors:** Fan Shi, Dehong Jiang, Junrong Ji, Jinsheng Yan, Huxing Chen

**Affiliations:** 1 School of Materials Science and Engineering, Zhejiang University, Hangzhou 310027, China; shifan@zju.edu.cn; 2 Chongzuo South Cement Co., Ltd., Chongzuo 532200, China; dehongjiang@163.com (D.J.); junrongji@163.com (J.J.); yanjinsheng2022@163.com (J.Y.)

**Keywords:** alkali, clinker, water-soluble hexavalent chromium

## Abstract

Due to the toxicity and mobility of chromium, the disposal of chromium-containing waste is a pressing issue. Co-processing of chromium-containing waste in a cement kiln is currently one of the most effective methods. However, the presence of water-soluble hexavalent chromium (Cr(VI)) in cement limits the use of this method. In this study, Na_2_CO_3_ was used to simulate alkali in industrial raw materials to investigate the pattern of influence of alkali content on water-soluble hexavalent chromium. The mechanisms associated with the oxidation and dissolution of chromium were investigated using X-ray diffraction (XRD), X-ray photoelectron spectroscopy (XPS), and inductively coupled plasma emission spectrometry (ICP-OES). The proportion of Cr(VI) in the clinker detected by XPS increased rapidly with increasing alkali content. In the cement slurry system, alkali promotes more hexavalent chromium leaching by influencing pH and other ion concentrations (Ca^2+^, SO_4_^2−^). Therefore, the addition of alkali to either the raw meal or to the cement slurry system will favour an increase in the water-soluble Cr(VI) content. This study may provide theoretical guidance for the preparation and use of clinkers containing chromium.

## 1. Introduction

Chromium (Cr), especially hexavalent chromium (Cr(VI)), is well known for its mutagenic and carcinogenic properties and is one of the three internationally recognised carcinogenic metals, as well as one of the 129 priority pollutants certified by the US EPA [1]. Chromium and its compounds are widely used in various areas of industrial production, but this has resulted in a large amount of chromium-containing solid waste, such as chromium slag and various chromium-containing sludges. Open piles of chromium-containing waste can leach chromium ions into the soil, groundwater, rivers, and lakes through rainwater washing, causing serious environmental pollution [2,3]. Exposure to Cr(VI) compounds in various forms can lead to respiratory cancers and other health damage [4,5]. Traditional disposal methods for solid waste containing chromium include landfill and incineration. Landfilling industrial waste not only takes up a lot of land resulting in a waste of land resources, but the chromium in the waste can easily contaminate soil and water [6]. Incineration is costly and inefficient, and causes secondary pollution. Reduction methods such as bioreduction, electrochemical reduction, and chemical reduction can also be used to treat some types of chromium-containing waste, but are often limited by high costs, high energy consumption, and the maximum concentration that can be disposed of [7,8]. Therefore, it is becoming increasingly important to find a safe and simple method of treating chromium-containing waste.

Co-processing in a cement kiln is one of the promising technologies for the disposal of chromium-containing solid waste as it can safely and effectively dispose of hazardous waste at low cost, while promoting the sustainable development of the cement industry [9]. Various types of waste (e.g., municipal solid waste, wastewater treatment plant sludge, waste plastics, scrap tires, and industrial wastes) co-processing in cement kilns has been widely and successfully implemented in developed countries [10,11]. However, the presence of Cr(VI) in cement can easily cause health hazards by leaching during cement worker operations. Chromate dermatitis is a recognised occupational disease in the construction industry. A study conducted by Avnstorp in Copenhagen, Denmark, showed that workers involved in the manufacturing of prefabricated concrete building components had the highest incidence of chromate sensitisation (10.5%) and hand eczema (11.9%) [12]. In 1983, Denmark passed legislation requiring that the water-soluble Cr(VI) levels in cement should not exceed 2 mg·kg^−1^ [13]. In June 2003, an EU Directive 2003/53/EC required that cement and cement product marketed and used should not contain more than 2 mg·kg^−1^ of water-soluble Cr(VI) [14]. The limit for water-soluble Cr(VI) in cement is 10 mg·kg^−1^ in China [15]. Consequently, how to control water-soluble Cr(VI) in cement is the key and bottleneck in the promotion of co-processing technology of chromium-containing waste in cement kilns. Currently, the standards are usually achieved by reducing water-soluble Cr(VI) ions to water-insoluble Cr(III) ions using reducing agents. Common reducing agents used in the cement industry include heptahydrate and monohydrate forms of ferrous sulphate, stannous sulphate, and Sb_2_O_3_. Reducing agents still have disadvantages, such as being prone to failure, affecting the performance of the cement, and being expensive [16,17]. For example, ferrous sulphate, one of the most widely used reducing agents, is susceptible to oxidation by air. In addition, there is evidence that ferrous sulphate affects the performance of cement and increases the corrosion of reinforcing steel in concrete [18]. Therefore, reducing agents cannot be added in large quantities. Factors affecting water-soluble Cr(VI) need to be investigated to improve the efficiency of cement kiln co-disposal of chromium-containing waste and to reduce the amount of reducing agent used.

The chromium in most chromium-containing wastes is dominated by Cr(III), but during the cement-burning process, Cr(III) can be oxidised to Cr(VI) in cement kilns under high-temperature, oxidising atmosphere and alkaline conditions [19]. Hence, the reduction of water-soluble Cr(VI) in cement can be achieved by both preventing the oxidation of Cr(III) to Cr(VI) and reducing the leaching of Cr(VI). There are many factors in the firing, grinding, and use of cement that affect the oxidation and leaching of chromium that have attracted the attention of many researchers. According to Linda Hills, the production of Cr(VI) in a kiln depends mainly on the oxygen and alkali content [20]. Increasing the partial pressure of oxygen favours the production of Cr(VI), while the reducing atmosphere effectively reduces the oxidation of chromium [21,22]. Cr(III) can be converted to Cr(VI) in the presence of alkali and alkaline earth metal oxides [23]. In a finishing mill, there are thermodynamically favourable conditions, such as high air volumes, gypsum dewatering moisture, grinding aids, and a strongly alkaline environment, for the cement. The alkali concentration is also important in the hydration phase, since the solubility of chromate, the main form of Cr(VI) present in the clinker, and the stable valence state of Cr are related to the pH of the filtrate [24,25].

Thus, alkali can influence the amount of water-soluble Cr(VI) in cement in many ways. The alkali in cement clinker mainly refers to two elements, potassium and sodium, such as Na_2_O, K_2_O and Na_2_CO_3_. Some alkali is inevitably brought in by raw materials and fuels during the cement production process. Many scholars have studied the effect of alkali on chromium from different aspects. Lee found that the water-soluble hexavalent chromium increased with increasing alkali content of the raw material [26]. The presence of alkali has been reported to accelerate chromium oxidation in studies on waste incineration, stainless steel slag treatment, and bushfires [27,28,29,30]. Therefore, whether alkali promotes the oxidation of chromium during clinker calcination needs to be explored. Magistri concluded that the soluble chromates are first immobilized in an ettringite-like structure phase, then released when the sulphate concentration in the solution increases [31]. Chrysochoou similarly concluded that SO_4_^2−^-ettringite is the most stable species and that sulphate can displace other oxygen anions, such as chromate, arsenate, and selenite [32]. As alkali content affects the solubility of sulphate [33], the effect of alkali on chromate is also worth investigating. However, the mechanism of the effect of alkali on water-soluble Cr(VI) in cement has not been fully investigated.

The main objective of this study was to determine the effect of alkali on water-soluble Cr(VI) during the firing and hydration stages and to provide theoretical guidance for the co-processing of solid waste containing chromium in cement kilns. For this purpose, a certain amount of chromium and different initial concentrations of alkali (Na_2_CO_3_) were added to the raw materials of cement. A quantitative study of the leaching of Cr(VI) was carried out using ultraviolet-visible spectrophotometry (UV-Vis). The mechanism of the effect of alkali on chromium in both the firing and leaching stages was further analysed using XRD, XPS, and ICP-OES.

## 2. Materials and Methods

### 2.1. Materials

Three Cr(III)-containing cement raw materials (A, B and C) were prepared using chemical-grade CaCO_3_, SiO_2_, A1_2_O_3_, Fe_2_O_3_, and C_6_H_9_CrO_6_, all of which have a mineral composition (calculated according to Bogue) close to that of common Portland cement. The proportions of the raw materials (A, B, and C) are presented in Table 1. The expected mineral phase compositions, according to the Bogue calculations, are also shown in Table 1 [34].

Fifteen samples were prepared by mixing A/B/C with different masses of chemical-grade Na_2_CO_3_ (Table 2). From A1 to A5 (or B1–B5, C1–C5), the sodium content was 0%, 0.5%, 1%, 2%, and 3% of the expected clinker mass (excluding H_2_O and CO_2_ from all raw materials), respectively. Each set of configured raw materials was mixed in a ball mill for 45 min, and then, the loss of ignition (LOI) was measured in three individual samples of the mixture to verify homogeneity. All samples were pressed into pellets and heated in a tube furnace at a rate of 5 °C per minute to 1400 °C and held for 45 min, with rapid cooling in air. The sintered pellets were crushed and mixed with 5 wt.% calcium sulfate dihydrate and then ground in a ball mill for 1 h.

### 2.2. Leaching Test

The leachability of hexavalent chromium is tested according to the standard method EN 196-10 [35]. This standard consists of two steps, extraction and analysis of the filtrate, and is applicable to the determination of water-soluble hexavalent chromium in cement. In order to obtain a homogeneous sample, it is treated according to EN 196-7 before the leaching test [36]. One part of cement, three parts of CEN standard sand, and one-half part of water (i.e., water/cement ratio 0.50) were mixed according to a fixed procedure for 270 s and then filtered to obtain the filtrate. The experiments in Section 3.3.2 also used acid or alkali solutions as elutants. The diluted filtrate was treated with s-diphenylcarbazide and acidified within a narrow range of pH. Chromium (VI) in acid solution forms a red-violet complex whose absorption is measured at 540 nm by UV-Vis (Sunny Hengping Instrument 722, CN). All tests were carried out within 2 h of obtaining the filtrate. The concentration (*C*) of Cr(VI) in the filtrate in mg/litre was determined from the calibration curve. The content of water-soluble Cr(VI) (*K*) in the cement was calculated by Formula (1).

(1) $$K=C\times \frac{V_{elutant}}{M_{cement}}$$

where *K* is the content of water-soluble Cr(VI) in the cement, in mg/Kg; *C* is the concentration of chromium (VI) in the filtrate, in mg/L; *V_elutant_* is the volume of elutant, in ml; and *M_cement_* is the mass of cement eluted, in g.

### 2.3. Analytical Methods

XRD was used to examine the crystalline composition of the material using an X-ray diffractometer (Shimadzu XRD-6000, Kyoto, Japan) equipped with a Cu Kα X-radiation. The valence changes of chromium in clinker samples are tested by XPS using an X-ray photoelectron spectrometer (Thermo Scientific K-Alpha, Waltham MA, USA). The measurements were carried out under an ultra-high vacuum. The data were calibrated for binding energy and fitted for analysis using Avantage software to determine the relative concentrations of the different chemical species. The concentrations of elements (Cr_Total_, Ca^2+^, Na^+^, SO_4_^2−^) in the leachate were determined using an inductively coupled plasma emission spectrometer (Agilent 720ES, Beijing, China).

## 3. Results and Discussion

### 3.1. Phase Evolution

**Figure 1:**
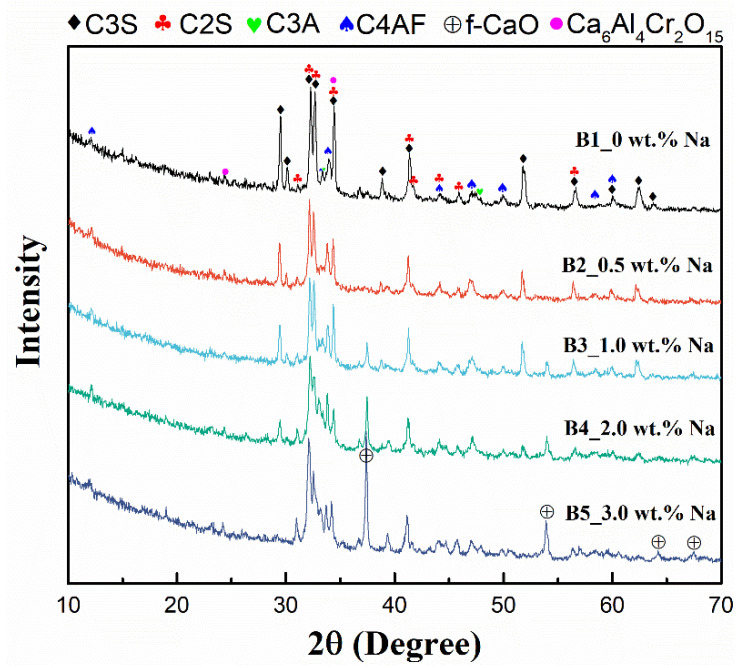
X-ray diffraction patterns of leach residues of samples B1–B5.

XRD was used to determine the changes in the composition of the mineral phases of the cement clinker after the addition of different masses of Na_2_CO_3_, and the results are shown in Figure 1. Samples A1–A5, B1–B5, and C1–C5 show similar patterns of evolution, so only B1–B5 are used here as an example to explain the results. According to the results, the main phase of the clinker B1–B5 was C_3_S, the second phase was C_2_S, and small amounts of C_3_A, C_4_AF and Cr(III)-containing phase Ca_6_Al_4_Cr_2_O_15_ were also present. With the addition of sodium (0.5 wt.%) to the raw material, the characteristic peak of f-CaO appeared, and as the sodium content increased from 0.5% to 3%, the characteristic peak of f-CaO became sharper, the characteristic peaks of C_3_S and Ca_6_Al_4_Cr_2_O_15_ gradually weakened, the characteristic peak of C_2_S increased, and the characteristic peaks of C_3_A and C_4_AF slightly increased. This indicates that as the sodium content increases from 0 to 3%, the contents of C_3_S and the Cr(III)-containing phase Ca_6_Al_4_Cr_2_O_15_ decrease, and the contents of C_2_S and free calcium oxide increase. This is due to the solid solution of sodium oxide in C_2_S and C_3_A, with mainly Na^+^ replacing Ca^2+^, the former preventing the reaction of C_2_S and f-CaO from forming C_3_S and the latter causing a change in the crystalline form of C_3_A (the variation was insignificant over the range of alkali content in this study), thus resulting in a lower C_3_S content. Due to the low content of both Cr and Na, XRD failed to detect more present phases. According to previous research, both Na and Cr have high solid solubility in the C_2_S phase, which is likely to facilitate the reaction of chromium and sodium to form sodium chromate [37]. The increase in f-CaO may facilitate the formation of calcium chromate [38]. Therefore, an increase in alkali content could affect the mineral composition of the clinker and, thus, the water-soluble Cr(VI).

### 3.2. Influence of the Alkali Content on the Total Cr(VI) in Cement

**Figure 2:**
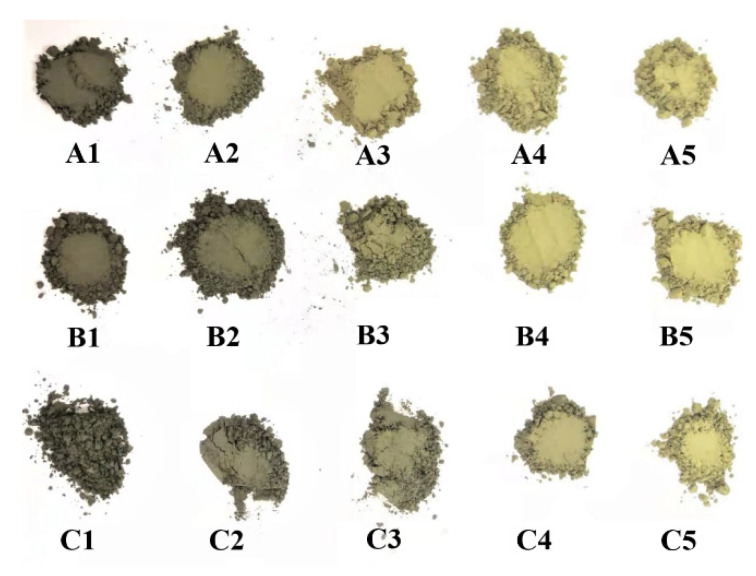
Samples (A1–A5, B1–B5 and C1–C5) after powdering.

The laboratory-prepared cement clinker after grinding is shown in Figure 2. In samples A1–A5, B1–B5, and C1–C5, the clinker colour gradually changed from dark green to yellow-green as the alkali content increased. Cr(III) oxide was dark green, and Cr(VI) oxide was yellow [39,40]. Thus, it is clear that the proportion of Cr(III) oxidised to Cr(VI) increases in samples A1–A5, B1–B5, and C1–C5.

**Figure 3:**
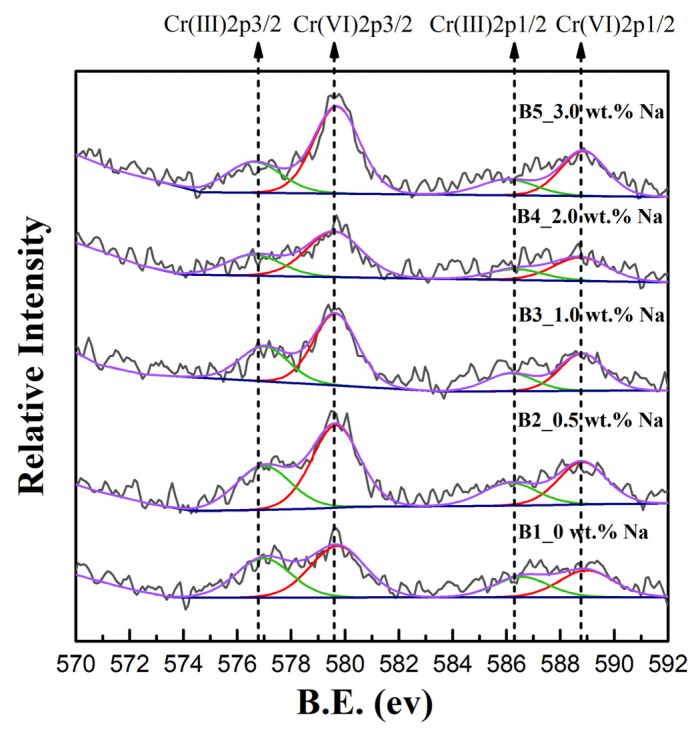
XPS spectra of the synthesized samples B1–B5.

The change in the valence state of chromium during firing was analysed by XPS. Samples A1–A5, B1–B5, and C1–C5 show similar patterns of evolution, so only the Cr 2p XPS spectra of samples B1–B5 are shown in Figure 3 as an example to explain the results. The presence of peaks associated with binding energies of 576.7, 579.7, 586.3, and 588.9 eV was identified by split peak fitting of the Cr 2p3/2 and Cr 2p1/2 photoelectron peaks. By comparing these binding energies with those of the standard spectra of Cr, the binding energies 586.3 ± 0.3 eV (Cr 2p1/2) and 576.7 ± 0.2 eV (Cr 2p3/2) are associated with Cr(III), while the binding energies 588.9 ± 0.2 eV (Cr 2p1/2) and 579.7 ± 0.1 eV (Cr 2p3/2) correspond to Cr(VI) [41]. When no sodium was added (B1), both Cr(III) and Cr(VI) were present in the clinker powder. When the sodium content increased from 0.5% to 3% (B2–B5), the area covered by the peaks corresponding to Cr(III) decreased, and the area covered by the peaks corresponding to Cr(VI) increased. Most of the chromium in sample B5 was present in the hexavalent form (Table 3). XPS analysis proved that the Cr(III) content decreased and the Cr(VI) content increased with increasing alkali content in samples B1–B5.

It has been reported that the presence of alkalis and alkaline oxides (e.g., K_2_O, Na_2_O) in an air atmosphere promoted the oxidation of Cr(III) to Cr(VI) [27,29,30]. Bram Verbinnen et al. demonstrated experimentally and by thermodynamic calculations that the reaction of Cr_2_O_3_ with oxygen alone in air to form CrO_3_ (Reaction 1, with a positive reaction Gibbs free energy) is not feasible below 1500 °C; the oxidation of Cr(III) is feasible in the presence of alkali or alkali metal oxides (Reactions 2–4, with negative reaction Gibbs free energies). Reactions involving NaOH and KOH have much lower Gibbs free energies than those involving CaO, and they are more likely to occur [23].

Cr_2_O_3_ + 3/2O_2_ → 2CrO_3_ (2)

Cr_2_O_3_ + 2CaO + 3/2O_2_ → 2CaCrO_4_ (3)

Cr_2_O_3_ + 4NaOH + 3/2O_2_ → 2Na_2_CrO_4_ + 2H_2_O (4)

Cr_2_O_3_ + 4KOH + 3/2O_2_ → 2K_2_CrO_4_ + 2H_2_O (5)

A similar reaction exists in the roasting of alkaline chromite:

(Fe,Mg)[Cr,Al,Fe]_2_O_4_ + 2Na_2_CO_3_ + 3O_2_ → 2Na_2_CrO_4_ + (Fe,Mg)[Al,Fe]_2_O_4_ + 2CO_2_ (6)

when a mixture of chromite ore and soda-ash is heated at 1200 °C, soluble sodium chromate can be produced [42].

Combining the experimental results of XPS with the above studies, the involvement of Na_2_CO_3_ in the oxidation of chromium during the firing of cement clinker can be described by the following equation:

Cr_2_O_3_ + 2Na_2_CO_3_ + 3/2O_2_ → 2Na_2_CrO_4_ + 2CO_2_ (7)

Therefore, the presence of alkali facilitates the oxidation of chromium by (1) participating directly in the oxidation reaction of chromium to produce sodium chromate and (2) increasing the f-CaO content to promote the formation of calcium chromate. As sodium chromate is much more soluble than calcium chromate, the generation of sodium chromate is also beneficial to increase the content of hexavalent chromium in the filtrate. In addition, alkali can lower the liquid-phase temperature to increase the liquid-phase volume and improve the diffusion rate and oxidation efficiency of chromium [43].

### 3.3. Influence of the Alkali Content on Hexavalent Chromium Leaching

**Figure 4:**
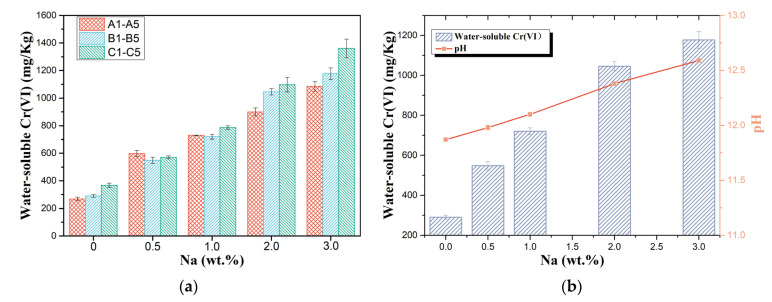
(**a**) Cr(VI) leached from samples A1–A5, B1–B5, and C1–C5, and (**b**) water-soluble Cr(VI) of samples B1–B5 and pH in the filtrates of samples B1–B5.

The concentration of water-soluble Cr(VI) in the leachate of each sample is shown in Figure 4a. It is worth stating that in order to study the mechanism of alkali influence on the oxidation as well as the dissolution of chromium in cement and to reduce the error, a higher amount of chromium and alkali was added to the raw materials, resulting in water-soluble hexavalent chromium and alkali content exceeding the limits of the relevant standards.

The concentration of hexavalent chromium in the filtrate increased rapidly with increasing alkali content, with consistent performance across the three different formulations of raw materials. In a horizontal comparison, when no sodium carbonate was added to the sample (A1, B1, and C1), the higher the percentage of C_2_S was, the lower the water-soluble Cr(VI) content. According to the literature, this may be because C_2_S is the phase with the highest amount of chromium solid solution among the four basic mineral phases of clinker [20,37]. However, when the sodium content was 0.5%, the samples with a high C_2_S percentage instead showed the highest increase in water-soluble Cr(VI) content. According to the conclusions in Section 3.2, this may be because Na^+^ is also preferentially soluble in C_2_S and Na^+^ promotes the oxidation of chromium and the formation of sodium chromate. As the alkali content increased, the proportion of chromium oxidised to hexavalent chromium in the clinker increased, and the increased sodium content also promoted the formation of the more soluble sodium chromate. At the same time, the pH of the filtrates was measured and displayed in Figure 4b. The pH of the filtrate also continued to rise after the addition of Na_2_CO_3_, with the same trend as the concentration of water-soluble hexavalent chromium. Considering that (1) the impact of alkali on cement hydration and the redox potential of chromium salts and (2) solution acidity and alkalinity affect solubility, the effect of alkali on chromium solubility in the leaching stage was further explored in terms of its influence on filtrate acidity and dissolution behaviour of chromium.

#### 3.3.1. Influence of Alkali on the Dissolution Behaviour of Chromium

**Figure 5:**
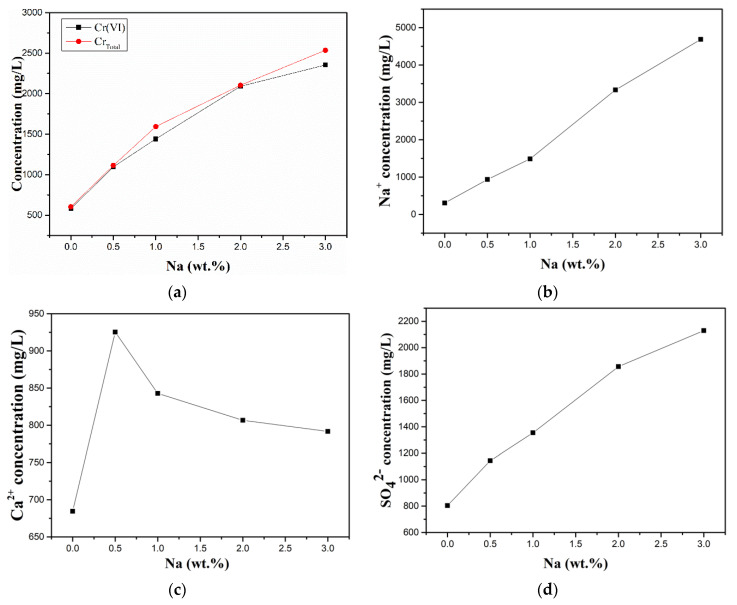
Concentrations of (**a**) Cr_Total_ and Cr(VI), (**b**) Na^+^, (**c**) Ca^2+^, and (**d**) SO_4_^2−^ in the filtrates of B1–B5.

To investigate the dissolution behaviour of chromium in cement slurry systems containing alkali, the concentrations of total chromium (Cr_Total_) and other related ions in the filtrates of B1 to B5 were tested by ICP-OES. As shown in Figure 5, the concentration of calcium ions in the filtrate increased and then decreased with increasing alkali content, while the contents of sodium ions, sulfate ions, and total chromium continued to increase. The increase in the content of sodium ions can be attributed to the increase in sodium carbonate content in the raw material. The increase in the content of calcium ions with the addition of 0.5 wt.% sodium is due to the alkali promoting the hydration of C_3_A and the dissolution of calcium sulfate dihydrate, but as the added amount increases, the pH of the filtrate also increases, resulting in a decrease in calcium ion concentration due to the formation of Ca(OH)_2_ precipitates. Owing to the limitation of the solubility product of calcium chromate, the decrease in the concentration of calcium ions increases the saturation concentration of chromate ions, which facilitates the dissolution of water-soluble hexavalent chromium (chromate) from cement.

The literature has shown that at the beginning of cement hydration, C_3_A can form a certain amount of chromate-containing ettringite in the presence of chromate (from the clinker itself). Simultaneously, as gypsum dissolves and the sulfate content of the solution increases, sulfate replaces the chromate in the ettringite to convert to the usually more stable sulfate ettringite, which allows the release of chromate ions from ettringite [31,32]. Figure 5d shows that the concentration of sulfate ions continues to increase with increasing alkali content. Alkali metals can increase the content of sulfate ions in the early solution by forming the better soluble alkali sulfates in the clinker [44] and by promoting gypsum dissolution (within the range of alkali content added in this study) [33], which may be one of the reasons for the elevated water-soluble hexavalent chromium content.

#### 3.3.2. Influence of pH of the Cement Slurry System

**Figure 6:**
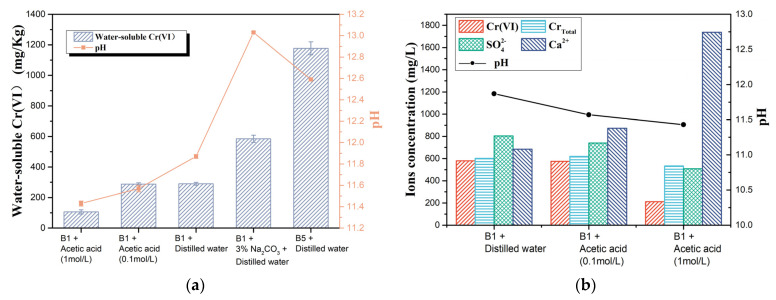
(**a**) Chromium concentration and pH of the filtrates of sample B1 with four kinds of extraction solvents and sample B5 and (**b**) concentrations of Cr_Total_, Cr(VI), Na^+^, Ca^2+^, and SO_4_^2−^ in the filtrates of B1 with three kinds of extraction solvents.

As the pH and hexavalent chromium concentrations in the filtrates of A1 to A5, B1 to B5, and C1 to C5 follow similar patterns, the correlation between them is a matter for consideration. To verify the effect of pH on chromate dissolution, five filtrates were obtained by extracting sample B1 (Na 0 wt.%) using four extractants with different pH levels (0.1 mol/L acetic acid, 1 mol/L acetic acid, distilled water, and Na_2_CO_3_ solution (Na mass equal to 3% of the clinker mass)) and sample B5 (Na 3 wt.%) using distilled water. The results of the pH and water-soluble Cr(VI) tests in the above five filtrates are shown in Figure 6a. The pH of the four filtrates of sample B1 was positively correlated with the pH of their extractants. Therefore, extraction with either an acid solution or an alkali solution can effectively affect the environment and pH of the cement slurry system.

The correlation between Cr(VI) content and filtrate pH in the four sets of filtrates of sample B1 was the same as in the previous test (Figure 4b). To further clarify the effect of pH of the cement slurry system on chromium dissolution, the concentrations of Cr_Total_, SO_4_^2−^, and Ca^2+^ in the filtrate extracted with deionised water and acid solutions were tested as shown in Figure 6b. As the pH of the filtrate decreased, the content of SO_4_^2−^, Cr(VI), and Cr_Total_ decreased and the content of Ca^2+^ and Cr(III) increased. The decrease in SO_4_^2−^ concentration reduces the dissolution of CrO_4_^2−^ (Section 3.3.1). Due to the limitation of the solubility product of calcium chromate, an increase in the concentration of calcium ions leads to a decrease in the saturation concentration of chromate ions, which also facilitates a reduction in the dissolution of water-soluble hexavalent chromium (chromate) from the cement. Therefore, the use of acid as a leaching agent to reduce the pH of the cement slurry system is beneficial in reducing the leaching of Cr(VI), but at the same time increases the leaching of Cr(III). The filtrate obtained with deionised water and B5 had a higher pH and Cr(VI) concentration compared to those obtained with B1 and deionised water. Therefore, the alkali in the cement clinker can affect the hexavalent chromium concentration by influencing the pH in the cement slurry system.

The filtrate obtained with Na_2_CO_3_ solution and B1 had a higher pH but lower Cr(VI) concentration compared to those obtained with B5 and deionised water, even though they had the same alkali content in the cement slurry system. Meanwhile, this phenomenon also suggested that influencing the content of Cr(VI) in the filtrate by affecting the pH of the cement slurry system is only one of the ways in which alkali affects water-soluble Cr(VI).

Repeated experiments using potassium carbonate as a source of alkali gave similar results to experiments using sodium carbonate in all tests (Figures S1–S3). Therefore, the effect of alkali on water-soluble Cr(VI) in cement is consistent in that it can participate in chromium oxidation reactions to promote oxidation as well as affect the filtrate environment to influence the leaching of hexavalent chromium.

## 4. Conclusions

The following conclusions can be drawn from this study.

1. The addition of alkali (sodium carbonate) to cement increases the amount of water-soluble hexavalent chromium in several ways. (1) The addition of alkali to the raw cement promotes the oxidation of trivalent chromium in the raw material, presumably by either increasing the amount of liquid phase or by participating in the oxidation reaction. (2) The alkali is involved in a reaction to produce sodium chromate which are more soluble than calcium chromate. (3) The alkali in cement promotes the dissolution of gypsum during hydration, allowing more of the chromate ions to be released from the calcium alumina by being replaced by sulfate ions. (4) It also increases the pH value of the cement slurry system, which improves the solubility of chromate and the stability of hexavalent chromium;
2. In summary, alkali affects the generation and dissolution of water-soluble hexavalent chromium in both the cement firing and initial hydration stages. Therefore, in cement production practice, especially when raw materials contain more trivalent chromium, attention should be given to controlling the content of alkali in raw materials. It is also important to control the total alkali content in the selection of cement admixtures and dopants.

## Figures and Tables

**Table 1:** Proportions and expected mineral composition of the raw materials.

Raw Materials	Proportions (wt.%)					Expected Clinker Mineral Composition (According to Bogue) (wt.%) ^2^			
	CaCO_3_	SiO_2_	A1_2_O_3_	Fe_2_O_3_	C_6_H_9_CrO_6_ ^1^	C_3_S ^3^	C_2_S ^4^	C_3_A ^5^	C_4_AF ^6^
A	76.24	14.31	3.89	2.72	2.84	55	23	9	13
B	76.56	14.00	3.89	2.71	2.84	60	18	9	13
C	77.20	13.40	3.87	2.70	2.83	70	8	9	13

^1^ The content of Cr is 1 wt.% of the raw material excluding H_2_O and CO_2_; ^2^ Calculated without considering the effect of chromium; ^3^ Tricalcium silicate (C_3_S); ^4^ dicalcium silicate (C_2_S); ^5^ tricalcium aluminate (C_3_A); ^6^ tetracalcium feroaluminate (C_4_AF).

**Table 2:** Composition of samples A1–A5, B1–B5 and C1–C5.

Samples	Proportions (wt.%)				Na Content in the Resulting Clinker (wt.%) (Excluding H_2_O and CO_2_ from All Raw Materials)
	A	B	C	Na_2_CO_3_	
A1	100.00	-	-	0	0
A2	99.26	-	-	0.74	0.5
A3	98.51	-	-	1.49	1.0
A4	97.03	-	-	2.97	2.0
A5	95.56	-	-	4.44	3.0
B1	-	100.00	-	0	0
B2	-	99.26	-	0.74	0.5
B3	-	98.52	-	1.48	1.0
B4	-	97.04	-	2.96	2.0
B5	-	95.56	-	4.44	3.0
C1	-	-	100.00	0	0
C2	-	-	99.26	0.74	0.5
C3	-	-	98.52	1.48	1.0
C4	-	-	97.05	2.95	2.0
C5	-	-	95.58	4.42	3.0

**Table 3:** XPS semiquantitative analysis of cement samples B1–B5.

Sample	Proportion of Chromium Species (at. %)	
	Cr(III)	Cr(VI)
B1	43.24	56.76
B2	37.89	62.11
B3	34.70	65.30
B4	31.54	68.46
B5	29.87	70.13

## Data Availability

Not applicable.

## Supplementary Materials

The following supporting information can be downloaded at: https://www.mdpi.com/article/10.3390/ijerph19084811/s1, Figure S1: Water-soluble Cr(VI) of samples B-K1–B-K5 and pH in the filtrates of samples B-K1–B-K5; Figure S2: X-ray diffraction patterns of leach residues of samples B-K1, B-K3 and B-K5; Figure S3. XPS spectra of the synthesized samples B-K1, B-K3 and B-K5.

## References

[1] Barceloux D.G., Barceloux D. (1999). Chromium. J. Toxicol. Clin. Toxicol..

[2] Loock-Hattingh M.M., Beukes J.P., van Zyl P.G., Tiedt L.R. (2015). Cr(VI) and Conductivity as Indicators of Surface Water Pollution from Ferrochrome Production in South Africa: Four Case Studies. Metall. Mater. Trans. B.

[3] Tumolo M., Ancona V., De Paola D., Losacco D., Campanale C., Massarelli C., Uricchio V.F. (2020). Chromium Pollution in European Water, Sources, Health Risk, and Remediation Strategies: An Overview. Int. J. Environ. Res. Public Health.

[4] Mishra S., Bharagava R.N. (2016). Toxic and genotoxic effects of hexavalent chromium in environment and its bioremediation strategies. J. Environ. Sci. Health Part C.

[5] Suh M., Wikoff D., Lipworth L., Goodman M., Fitch S., Mittal L., Ring C., Proctor D. (2019). Hexavalent chromium and stomach cancer: A systematic review and meta-analysis. Crit. Rev. Toxicol..

[6] Huang W., Fooladi H. (2021). Economic and environmental estimated assessment of power production from municipal solid waste using anaerobic digestion and landfill gas technologies. Energy Rep..

[7] Xia S., Song Z., Jeyakumar P., Shaheen S.M., Rinklebe J., Ok Y.S., Bolan N., Wang H. (2019). A critical review on bioremediation technologies for Cr(VI)-contaminated soils and wastewater. Crit. Rev. Environ. Sci. Technol..

[8] Yuan L., Xu X., Li H., Wang Q., Wang N., Yu H. (2017). The influence of macroelements on energy consumption during periodic power electrokinetic remediation of heavy metals contaminated black soil. Electrochim. Acta.

[9] Wang Y., Zhou H., Jiang X. (2018). Research situation and development of co-processing of hazardous waste in cement kiln. Environ. Pollut. Control.

[10] Pavlík Z., Fořt J., Záleská M., Pavlíková M., Trník A., Medved I., Keppert M., Koutsoukos P.G., Černý R. (2016). Energy-efficient thermal treatment of sewage sludge for its application in blended cements. J. Clean Prod..

[11] Xiao H., Li Y., Wang M., Yan D., Liu Z. (2021). The migration and transformation of chromium during co-processing of cement raw meal mixed with chrome-polluted soil. Environ. Technol. Innov..

[12] Avnstorp C. (1991). Risk factors for cement eczema. Contact Dermat..

[13] (1984). Cement-Water Soluble Chromate-Test Method.

[14] European Union (2003). Directive 2003/53/EC of the European Parliament and of the Council of 18 June 2003 amending for the 26th time Council Directive 76/769/EEC relating to restrictions on the marketing and use of certain dangerous substances and preparations (nonylphenol, nonylphenol ethoxylate and cement). Off. J. Eur. Union.

[15] (2015). Limit and Determination of the Water-Soluble Chromium (VI) Content for Cement.

[16] Erdem E., Güngörmüş H., Kılınçarslan R. (2016). The investigation of some properties of cement and removal of water soluble toxic chromium(VI) ion in cement by means of different reducing agents. Constr. Build. Mater..

[17] Magistri M., D’Arcangelo P. (2008). New chromium reducing agent for cement. ZKG Int..

[18] Roskovic R., Stipanovic Oslakovic I., Radic J., Serdar M. (2011). Effects of chromium(VI) reducing agents in cement on corrosion of reinforcing steel. Cem. Concr. Compos..

[19] Frias M., Sánchez de Rojas M.I. (2002). Total and soluble chromium, nickel and cobalt content in the main materials used in the manufacturing of Spanish commercial cements. Cem. Concr. Res..

[20] Hills L., Johansen C.V. (2007). Hexavalent Chromium in Cement Manufacturing: Literature Review.

[21] Chen Y.L., Lai Y.C., Lin C.J., Chang Y.K., Ko M.S. (2013). Controlling sintering atmosphere to reduce the hazardous characteristics of low-energy cement produced with chromium compounds. J. Clean Prod..

[22] Liu D., Diao J., Qiu Y., Wang G., Li G., Xie B. (2020). Determination of chromium valence state in the CaO–SiO_2_–FeO–MgO–CrO_x_ system by X-ray photoelectron spectroscopy. High Temp. Mater. Processes.

[23] Verbinnen B., Billen P., Van Coninckxloo M., Vandecasteele C. (2013). Heating Temperature Dependence of Cr(III) Oxidation in the Presence of Alkali and Alkaline Earth Salts and Subsequent Cr(VI) Leaching Behavior. Environ. Sci. Technol..

[24] Bhatty J.I., Miller F.M., West P.B., Ost B.W. (1999). Stabilization of Heavy Metals in Portland Cement, Silica Fume/Portland Cement and Masonry Cement Matrices.

[25] Estokova A., Palascakova L., Kanuchova M. (2018). Study on Cr (VI) leaching from cement and cement composites. Int. J. Environ. Res. Public Health.

[26] Kyu L.J., Hun S. (2011). Leaching Properties of Water-Soluble Hexavalent Chromium by Manufacturing Condition of Cement Clinker. Korean J. Mater. Res..

[27] Zhao Q., Liu C., Cao L., Zheng X., Jiang M. (2018). Effect of Lime on Stability of Chromium in Stainless Steel Slag. Minerals.

[28] Panichev N., Mabasa W., Ngobeni P., Mandiwana K., Panicheva S. (2008). The oxidation of Cr(III) to Cr(VI) in the environment by atmospheric oxygen during the bush fires. J. Hazard. Mater..

[29] Hu H., Xu Z., Liu H., Chen D., Li A., Yao H., Naruse I. (2015). Mechanism of chromium oxidation by alkali and alkaline earth metals during municipal solid waste incineration. Proc. Combust. Inst..

[30] Lehmusto J., Lindberg D., Yrjas P., Skrifvars B.J., Hupa M. (2012). Thermogravimetric studies of high temperature reactions between potassium salts and chromium. Corros. Sci..

[31] Magistri M., Cerulli T., Padovani D., Cella F., Presti A.L. The Effect of cement hydration on the release mechanism of soluble chromates. Proceedings of the Conference ICCC.

[32] Chrysochoou M., Dermatas D. (2006). Evaluation of ettringite and hydrocalumite formation for heavy metal immobilization: Literature review and experimental study. J. Hazard. Mater..

[33] Yuan T., Wang J., Li Z. (2010). Measurement and modelling of solubility for calcium sulfate dihydrate and calcium hydroxide in NaOH/KOH solutions. Fluid Phase Equilibria.

[34] Harrisson A.M., Hewlett P.C., Liska M. (2019). 4-Constitution and Specification of Portland Cement. Lea’s Chemistry of Cement and Concrete.

[35] (2016). Methods of Testing Cement-Part 10: Determination of the Water-Soluble Chromium (VI) Content of Cement.

[36] (2007). Methods of Testing Cement-Part 7: Methods of Taking and Preparing Samples of Cement.

[37] Barros A.M., Espinosa D.C.R., Tenório J.A.S. (2004). Effect of Cr_2_O_3_ and NiO additions on the phase transformations at high temperature in Portland cement. Cem. Concr. Res..

[38] Yang Y., Ma H., Chen X., Zhu C., Li X. (2020). Effect of incineration temperature on chromium speciation in real chromium-rich tannery sludge under air atmosphere. Environ. Res..

[39] Wu Y., Song S., Garbers-Craig A.M., Xue Z. (2018). Formation and leachability of hexavalent chromium in the Al_2_O_3_-CaO-MgO-Cr_2_O_3_ system. J. Eur. Ceram. Soc..

[40] Heerah M.Z., Galobardes I., Dawson G. (2021). Characterisation and control of cementitious mixes with colour pigment admixtures. Case Stud. Constr. Mater..

[41] (2000). NIST X-ray Photoelectron Spectroscopy Database.

[42] Antony M.P., Tathavadkar V.D., Calvert C.C., Jha A. (2001). The soda-ash roasting of chromite ore processing residue for the reclamation of chromium. Metall. Mater. Trans. B.

[43] Herfort D., Macphee D.E., Hewlett P.C., Liska M. (2019). 3-Components in Portland Cement Clinker and Their Phase Relationships. Lea’s Chemistry of Cement and Concrete.

[44] Fregert S., Gruvberger B. (1973). Correlation between alkali sulphate and water-soluble chromate in cement. Acta Derm.-Venereol..

