# Supplementary material for: Effects of Alkali on Water Soluble Hexavalent Chromium in Ordinary Portland Cement
Source: Int J Environ Res Public Health. 2022 Apr 15;19(8):4811. doi: 10.3390/ijerph19084811 (PMC9025607; doi:10.3390/ijerph19084811)
Supplement: Supplementary file 1 [file ijerph-19-04811-s001.zip › ijerph-1604628-Supplementary Figures.pdf]

Supplementary Figures

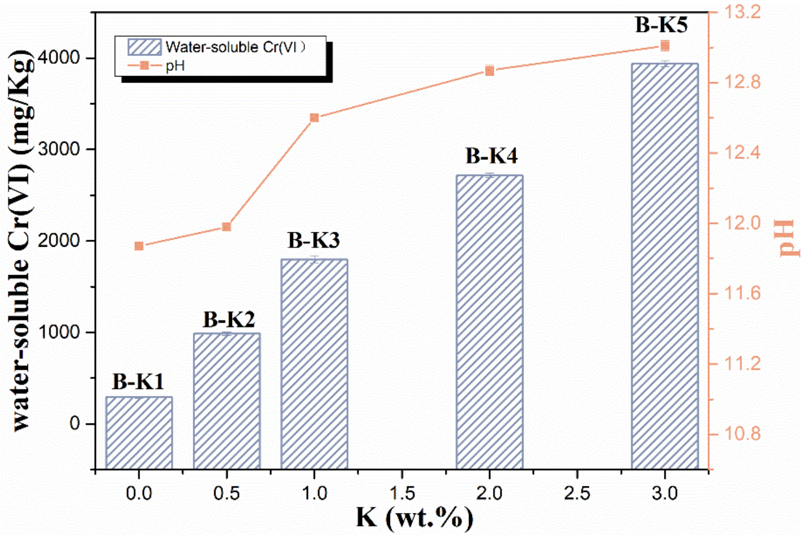

Figure S1. Water-soluble Cr(VI) of samples B-K1–B-K5 and pH in the filtrates of samples B-K1–B-K5.

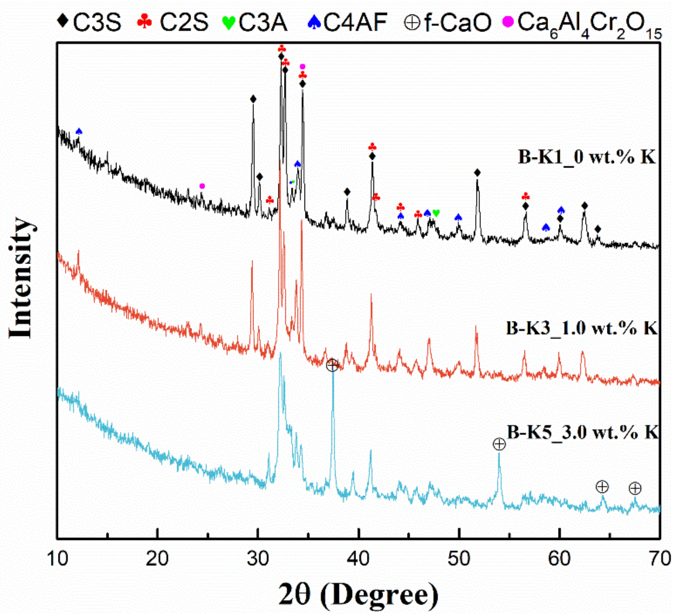

Figure S2. X-ray diffraction patterns of leach residues of samples B-K1, B-K3 and B-K5.

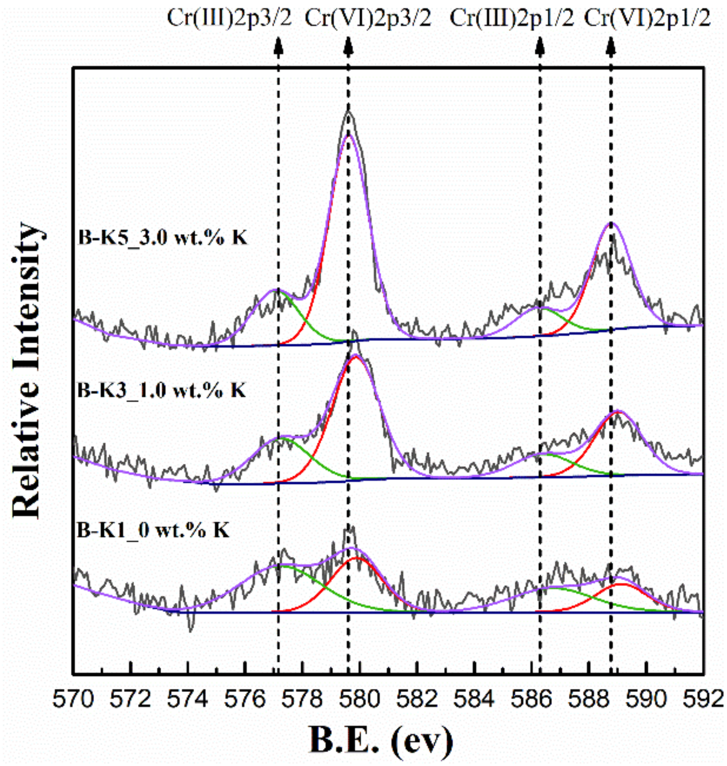

Figure S3. XPS spectra of the synthesized samples B-K1, B-K3 and B-K5.
